# Interpretations of Frequency Domain Analyses of Neural Entrainment: Periodicity, Fundamental Frequency, and Harmonics

**DOI:** 10.3389/fnhum.2016.00274

**Published:** 2016-06-06

**Authors:** Hong Zhou, Lucia Melloni, David Poeppel, Nai Ding

**Affiliations:** ^1^College of Biomedical Engineering and Instrument Sciences, Zhejiang UniversityHangzhou, China; ^2^Department of Neurology, New York University Langone Medical CenterNew York, NY, USA; ^3^Department of Neurophysiology, Max-Planck Institute for Brain ResearchFrankfurt, Germany; ^4^Neuroscience Department, Max-Planck Institute for Empirical AestheticsFrankfurt, Germany; ^5^Department of Psychology, New York UniversityNew York, NY, USA; ^6^Interdisciplinary Center for Social Sciences, Zhejiang UniversityHangzhou, China; ^7^Neuro and Behavior EconLab, Zhejiang University of Finance and EconomicsHangzhou, China

**Keywords:** neural entrainment, rhythm, harmonics, oscillations, periodicity

## Abstract

Brain activity can follow the rhythms of dynamic sensory stimuli, such as speech and music, a phenomenon called neural entrainment. It has been hypothesized that low-frequency neural entrainment in the neural delta and theta bands provides a potential mechanism to represent and integrate temporal information. Low-frequency neural entrainment is often studied using periodically changing stimuli and is analyzed in the frequency domain using the Fourier analysis. The Fourier analysis decomposes a periodic signal into harmonically related sinusoids. However, it is not intuitive how these harmonically related components are related to the response waveform. Here, we explain the interpretation of response harmonics, with a special focus on very low-frequency neural entrainment near 1 Hz. It is illustrated why neural responses repeating at *f* Hz do not necessarily generate any neural response at *f* Hz in the Fourier spectrum. A strong neural response at *f* Hz indicates that the time scales of the neural response waveform within each cycle match the time scales of the stimulus rhythm. Therefore, neural entrainment at very low frequency implies not only that the neural response repeats at *f* Hz but also that each period of the neural response is a slow wave matching the time scale of a *f* Hz sinusoid.

## Introduction

Cortical activity, measured by electroencephalography (EEG), magnetoencephalography (MEG), or local field potential recordings (LFP), can synchronize to the rhythm of a sensory stimulus. For example, when the intensity of a sound, e.g., a pure tone, fluctuates at a given frequency (*f* Hz), a neural response at that frequency (*f* Hz) is often observed and is referred to as the auditory steady state response (aSSR; Galambos et al., 1981; Ross et al., 2000; Wang et al., 2012). Similarly, when the luminance of a visual stimulus, e.g., a Gabor patch, fluctuates at *f* Hz, a neural response at *f* Hz can also be observed and is referred to as the steady state visual evoked response (SSVEP; Norcia et al., 2015). Recently, low-frequency (<3 Hz) neural entrainment has also been observed for abstract stimulus properties such as the rhythms of musical beats and linguistic constituents (Buiatti et al., 2009; Nozaradan et al., 2011; Ding et al., 2016) and during the processing of natural speech or movies (Ding and Simon, 2012; Zion Golumbic et al., 2013; Koskinen and Seppä, 2014; Lankinen et al., 2014). It has been hypothesized that low-frequency neural synchronization to a stimulus provides a mechanism for selective attention and temporal integration of information (Schroeder et al., 2008; Schroeder and Lakatos, 2009; Giraud and Poeppel, 2012), and is important for parsing the temporal structure of speech and music (Nozaradan et al., 2011; Ding et al., 2016).

Neural entrainment to a stimulus rhythms is often analyzed in the frequency domain, while traditional neurophysiological responses, e.g., the event-related responses, are usually analyzed in the time domain. Therefore, some frequency-domain measures may appear unintuitive for researchers mainly using time domain analysis methods. For example, when the stimulus rhythm is at *f* Hz, neural responses can often be observed not just at *f* but also at its harmonics, i.e., at 2*f*, 3*f*, 4*f* etc. The harmonics can provide additional insight into the underlying neural encoding mechanisms (O’connell et al., 2015) but their interpretations are not straightforward. In this article, we explain how these harmonics are related to time-domain waveforms. We restrict the frequency-domain analysis method to the Discrete Fourier Transform (DFT), the most classic frequency domain analysis method. We will elaborate how the properties of a signal are represented in the lens of the DFT, and will not discuss whether the DFT is the best method to represent a particular signal.

The article is organized as follows: we first present examples that describe the relationship between time-domain signal periodicity and signal spectrum. Since it remains controversial whether the experimentally observed neural tracking of such low-frequency stimulus rhythms reflects a succession of event-related responses or a proper entrainment of neural oscillators (Ding and Simon, 2014; Keitel et al., 2014). We also describe how to interpret the power spectrum of a series of event-related responses. These discussions are purely based on intuitive examples, avoiding a more formal, mathematical treatment (for formal treatment, see, e.g., Oppenheim et al., 1989). A glossary is provided in Table 1.

**Table 1:** **Glossary table**.


**Basic definitions**
Signal measurement	In principle, a signal could be infinite in duration. In practice, we can only measure a signal in a finite interval, e.g., from *T*_0_ to *T*_1_. The signal measured in this finite interval is called a measurement.
Periodic signal	A signal *x*(*t*) is periodic if *x*(*t*) = *x*(*t* + *T*), for any *t*.
	If *x*(*t*) = *x*(*t* + *T*), then it follows that *x*(*t* + *kT*) = *x*(*t*) for any integer *k*. A periodic signal is infinite in duration and in this article, we only consider a measurement that has a duration of *kT*.
Period	The period of a signal is *T* if and only if *T* the smallest positive number such that *x*(*t*) = *x*(*t* + *T*).
Fundamental frequency	The fundamental frequency of a signal is *f*_0_ = 1/*T*, where *T* is the period of the signal.
Harmonics	The frequency of the *k*th harmonic is *k* times the fundamental frequency, i.e., *kf*_0_.
Discrete-time signals	A continuous-time signal *x*(*t*) can be converted into a discrete-time signal by a sampling process. If the sampling period is *t*_s_, the discrete signal measurement will be *x*(*kt*_s_), where *k* is an integer and *T*_0_	#x0003C; *kt*_s_	#x0003C; *T*_1_.
Sampling frequency	If the sampling period is *t*_s_, the sampling frequency is *f*_s_ = 1/*t*_s_.
**Discrete Fourier Transform (DFT)**	
DFT	The DFT represents a discrete-time signal as the sum of harmonically related sinusoids. The DFT coefficients determine the amplitude and phase of these sinusoids.
Frequency extracted by the DFT	If a discrete-time signal measurement contains *D* samples, i.e., time points, the Fourier transform represents it as the sum of sinusoids at harmonically related frequencies, i.e., *k*/*D*, where *k* is an integer and *k*/*D* falls between 0 and half of the sampling rate.
Signal extrapolation	The DFT assumes that a signal measurement constitutes a single period of a periodic signal. In other words, the DFT extrapolates the signal measurement by repeating the same waveform in time. For a periodic signal, such extrapolation is correct as long as an integer number of periods of the signal are measured.
**Neuroscience terms**	
Event-related responses	The event-related response refers to the response waveform precisely following, i.e., time-locked (often called phase-locked) to, a brief sensory stimulus. To measure the event-related response, a brief stimulus is presented repetitively for tens or hundreds of times and the interval between two presentations is usually randomized. The event-related response is obtained by averaging the response waveform over repetitions of the stimulus.
High-gamma activity	Neural activity in the high-gamma band, which is usually defined to be between 70 and 200 Hz. The waveform of high-gamma activity is usually not synchronized to the sensory stimuli while the power envelope of high-gamma activity is sometimes synchronized to sensory stimuli.

## Signal Periodicity and the Fourier Spectrum

**Figure 1:**
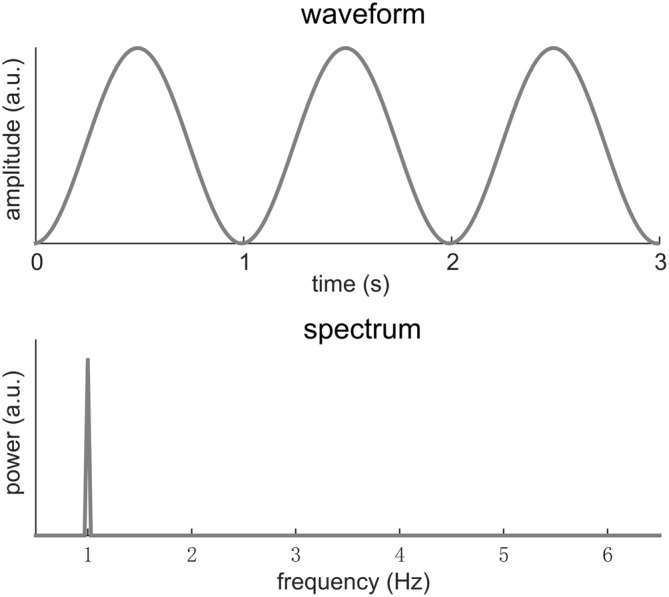
**A 1-Hz sinusoid.** In the spectrum, the signal power concentrates at 1 Hz. Here, we only focus on the shape of the neural response waveform rather than its scale. Therefore the amplitude is of an arbitrary unit (a.u.).

Here we will consider a signal that has a period of *T*. In other words, the signal repeats every *T* seconds. The Fourier transform analyses the frequency content of a signal by decomposing it into sinusoids at different frequencies. A signal with a period *T* repeats at a rate of *f*_0_ = 1/*T*, which is referred to as the fundamental frequency of the signal. Usually and intuitively, the Fourier spectrum of such a signal shows strong power at *f*_0_. In other words, the signal can be well explained by a sinusoid at frequency *f*_0_. Sometimes, the response at *f*_0_ may be the only component in the Fourier spectrum, indicating that the signal is a sinusoid. Figure 1 illustrates this condition.

**Figure 2:**
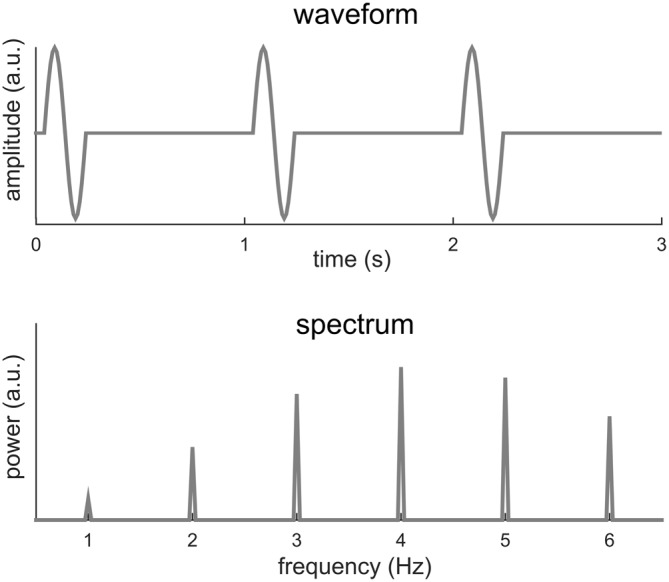
**One cycle of a 5-Hz sinusoid repeating every 1 s.** The spectrum of the signal shows power at multiple frequencies, at both the fundamental frequency, i.e., 1 Hz, and harmonics, i.e., multiples of 1 Hz. The strongest power appears at the 4th harmonic rather than at the fundamental frequency.

Sinusoids, of course, are mathematical abstractions and in the real world few signals are precisely sinusoidal. When a signal deviates from a sinusoid, its Fourier spectrum will have power not just at *f*_0_, but also at multiples of *f*_0_, e.g., 2*f*_0_, 3*f*_0_, 4*f*_0_ etc. Figure 2 illustrates one such condition, in which a short biphasic signal lasting for 200 ms repeats every 1 s. In this illustration, the biphasic signal is one cycle of a 5 Hz sinusoid while the signal repeats at 1 Hz (i.e., *f*_0_ = 1 Hz). The power spectrum of this signal spreads over a number of frequencies, e.g., *f*_0_, 2*f*_0_, 3*f*_0_, 4*f*_0_… The strongest power in the Fourier spectrum appears at 4*f*_0_ instead of *f*_0_. This signal can be viewed as a rough simulation of a sequence of transient event-related responses.

**Figure 3:**
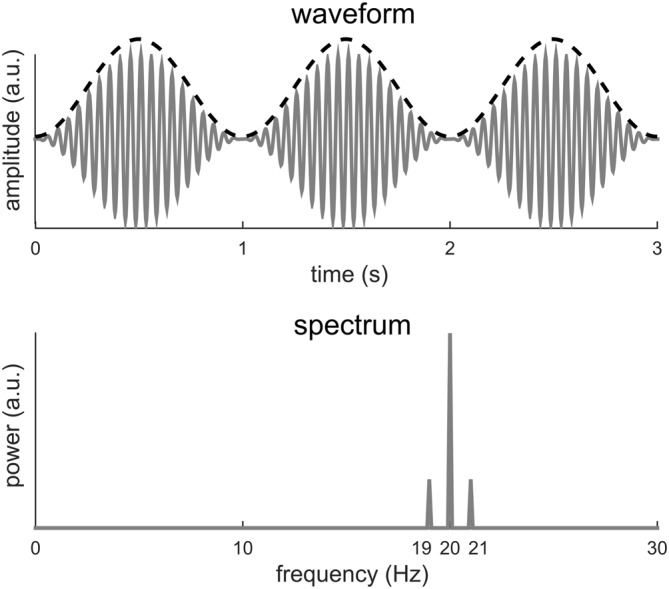
**A 20-Hz sinusoid amplitude modulated at 1 Hz.** The dashed black curve shows the envelope and the gray curve shows the waveform. The 1-Hz envelope imposes a clear 1 Hz rhythm in how the signal power fluctuates in time. The spectrum of the modulated signal, however, shows no power at 1 Hz but instead power at 20 Hz and 20 ± 1 Hz.

Figure 2 illustrates that a signal with a period *T* may have its power spread over *f*_0_ and its harmonics. In the following, we show an additional example in which no power even exists at *f*_0_. In this example (Figure 3), a 10-Hz sinusoid is amplitude modulated at 1 Hz. Amplitude modulation involves the product of two signals. The fast signal is called the carrier and the slow signal is called the envelope. In general, the envelope captures how the signal power fluctuates over time. In Figure 3, the modulated signal is the product of a 20-Hz sinusoid and a 1-Hz sinusoid. The amplitude modulated signal has a period of 1 s, as is evident from its waveform. Nonetheless, the Fourier spectrum shows no power at 1 Hz. In this example, the envelope signal is a sinusoid, if the signal is not a sinusoid, additional responses will be seen at 20 ± 2 Hz, 20 ± 3 Hz…, on top of the responses at 20 Hz and 20 ± 1 Hz.

**Figure 4:**
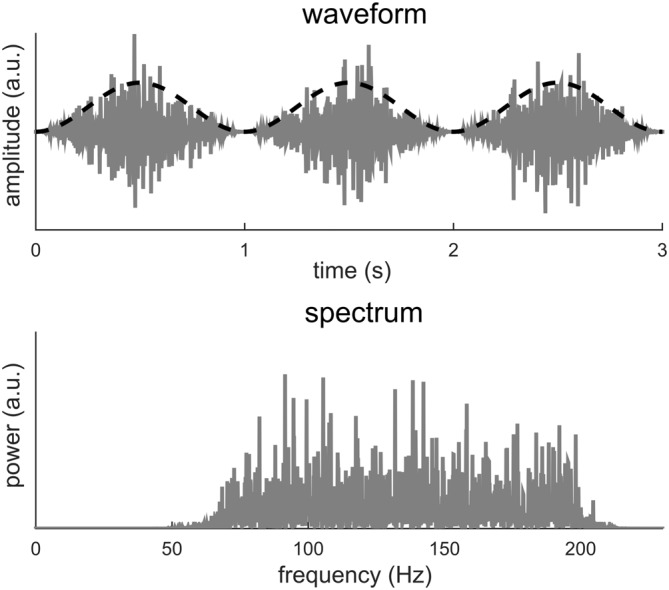
**A broadband noise between 70 and 200 Hz is amplitude modulated at 1 Hz.** The dashed black curve shows the envelope while the gray curve shows the waveform. The 1-Hz envelope imposes a clear 1 Hz rhythm in how the signal power fluctuates in time. The spectrum of the modulated signal, however, shows no power at 1 Hz.

The example in Figure 3 also introduces more general cases in which signal periodicity occurs in the modulation domain, i.e., in the signal envelope. In Figure 4, the carrier is a band-limited white noise between 70 and 200 Hz while the envelope signal remains a 1 Hz sinusoid. A visual inspection of the signal waveform suggests a strong rhythm at 1 Hz while no 1 Hz information can be found in the spectrum. In this case, the apparent 1 Hz periodicity only exists in the signal envelope and can only be revealed after the envelope signal by itself is extracted. A Fourier analysis of the envelope reduces to the condition illustrated in Figure 1. This example can be viewed as a simulation of high-gamma neural activity tracking a 1-Hz rhythm. The signal envelope can be extracted either explicitly using, e.g., the Hilbert transform, or implicitly through a time-frequency analysis, such as the short-term Fourier transform (STFT) or the wavelet transform. One interpretation of the spectrogram obtained by the STFT or wavelet analysis is that the input signal is filtered into narrow frequency bands and in each band the power envelope of the signal is extracted (Vaidyanathan, 1990). Therefore, periodicity in the modulation domain can be revealed by analyzing the time course of the STFT or wavelet spectrogram.

In sum, we show here that if the period of a signal is *T*, the DFT spectrum of the signal may show power at *f*_0_ and its harmonically related frequencies. Importantly, the power at *f*_0_ may not be the strongest (Figure 2) and may not even exist (Figure 3). Furthermore, even when the signal is a periodic, it may show higher order regularity including the periodicity in its envelope (Figure 4).

## Factors Affecting the Power at Harmonic Frequency

**Figure 5:**
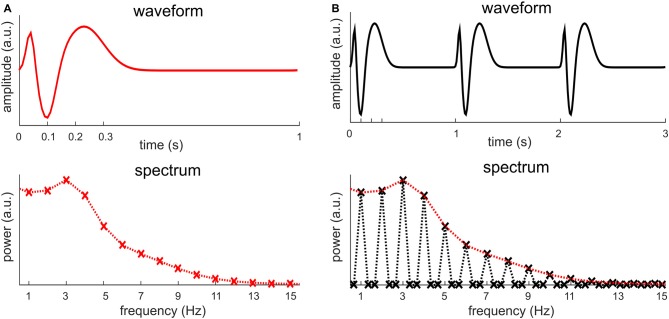
**The relationship between the spectrum of a periodic signal and the spectrum of a single cycle. (A)** The waveform and spectrum of one signal cycle, which can be viewed as a simulation of an event-related response. The signal measurement lasts for 1 s while the signal only fluctuates for about 300 ms. When the signal measurement is 1 s, the spectrum only shows discrete values at 1 Hz and its harmonics, marked by a cross. It is customary, however, to connect the discrete values as a curve, shown by the dotted curve. **(B)** When the signal in **(A)** repeats every 1 s, it construct a periodic signal. The figure shows a 3-s measurement of the periodic signal, which includes three cycles of the signal^1^. The spectrum of this signal shows discrete values at 1/3 Hz and its harmonics. The power is zero, however, at frequencies that are not the harmonics of 1 Hz, i.e., the fundamental frequency of the signal. The spectrum in **(A)** is reproduced in **(B)**, in red. It is clear that the spectrum in **(A)** is the envelope of the spectrum of the signal in **(B)**.

The previous section shows that a neural signal repeating every *T* seconds is represented in the frequency domain by harmonically related frequencies at *f*_0_, 2*f*_0_, 3*f*_0_, 4*f*_0_… In this section, we discuss in more details about what factors decide the power at each frequency. A periodic signal is fully characterized by a single cycle. Figure 5 illustrates the Fourier analysis of a periodic signal (including multiple cycles) and the Fourier analysis of a single cycle. All non-zero values in the spectrum of a periodic signal are captured by the spectrum of a single cycle. The spectrum of a periodic signal can be obtained by inserting zeros into the spectrum of a single cycle. The spectrum of a periodic signal can only have nonzero values at the fundamental frequency and its harmonics, and the spectrum of a single cycle only takes values at these frequencies. In general, the non-zero values in the spectrum of a periodic signal are decided by the waveform of a single cycle while the frequencies at which the spectrum shows nonzero values are decided by its period. In other words, the spectrum of a single cycle provides the spectral envelope of the spectrum of a periodic signal.

**Figure 6:**
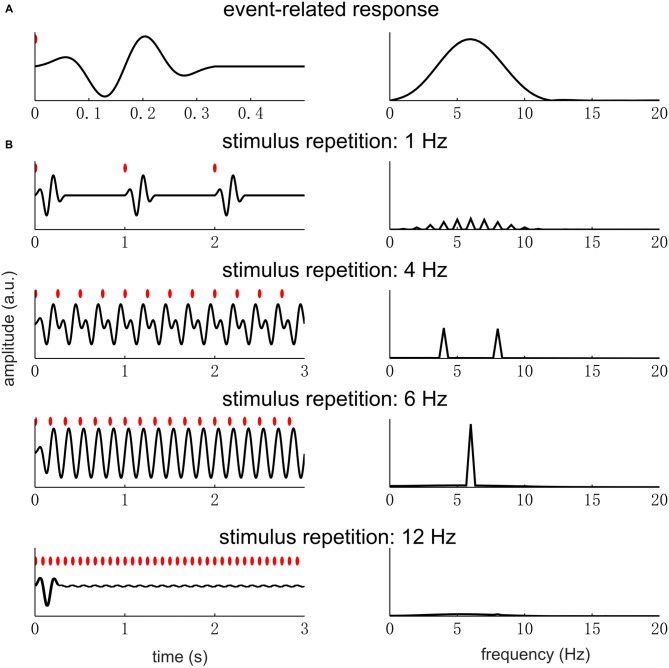
**Event-related response repeating at different rates.** The left panel figures show the response waveform while the right panel figures show the response spectrum. In the left panel figures, each red dot indicates a sensory event. **(A)** A single event-related response, whose power concentrates in the theta band (4–8 Hz). **(B)** The responses to sensory events repeating at different rates. When the stimulus rate is far below the theta band, e.g., at 1 Hz, there is little overlap between the responses to different sensory events. In the spectrum, the response power is weak and distributed over a number of harmonically related frequencies. When the stimulus is within the theta band, e.g., 4 and 6 Hz, the responses to different sensory events overlap. In the spectrum, a strong response is seen at the fundamental frequency of the stimulus rhythm and also at the second harmonic if it falls in the resonance frequency range of the event-related response. If the stimulus rate is very high, a clear response only appears at the stimulus onset.

Based on the analysis above, when the waveform of a single cycle contains “fast” oscillations or “sharp” edges, the signal will have high power at high-frequency harmonics. Here, “fast” oscillation means oscillations at frequencies much higher than the fundamental frequency of the periodic signal (Figure 3). “Sharp” edges mean edges rising/decaying faster relative to how fast a sinusoid at *f*_0_ rises or decays. The power of a periodic signal will concentrate at *f*_0_ only if the stimulus rate matches the spectral resonance, i.e., time scales, of the response in a single stimulus cycle (for details see, the next section and Figure 6). Therefore, a neural peak at *f*_0_ Hz in the Fourier spectrum does not only indicate the repetition of a neural waveform at *f*_0_ Hz but also indicates that the waveform being repeated is roughly a cycle of a *f*_0_ Hz sinusoid.

## Fourier Analysis of a Series of Event-Related Responses

In this section, we describe the frequency domain representation of a periodic neural response is composed of a series of discrete, steady-state event-related responses. Specifically, the sensory stimulus is a sequence of periodically occurring events and each event is modeled as an impulse, which can be viewed as an approximation of a short tone pip or a short flash of light. We assume that, when the neural response reaches steady state, the event-related response to each sensory event is identical (time-invariance), and the measured neural response is a linear superposition of different event-related responses (linearity). Under these assumptions, the neural network generating the measured neural response can be modeled by a linear time-invariant system (Oppenheim et al., 1989). It then follows that intrinsic properties of the neural network are fully characterized by the impulse response, i.e., the event-related response.

Under the linear time-invariant theory, each cycle of the neural response, i.e., the event-related response, is a property of the neural system while periodicity is a property of the stimulus. If the event-related response decays to baseline within each stimulus cycle, the spectral envelope of the periodic neural response is determined by the spectrum of the event-related response (Figure 5). This conclusion, however, also holds if the event-related response does not return to baseline when the next stimulus comes for reasons that will not be elaborated^2^. Furthermore, the linear time-invariant system model also applies to aperiodic stimuli and to continuously changing stimuli. This article, however, only focuses on the response to a periodic sensory input.

In Figure 6, we illustrate how the spectrum of a single event-related response and the stimulus repetition rate jointly determine the spectrum of the neural responses under the linear time-invariant systems theory. In this example, we assume that the event-related response has the strongest power in the theta band (4–8 Hz) and the stimulus is a sequence of pulses, i.e., very brief sensory events. The resonance frequency range of the neural system, i.e., the theta band, is arbitarily chosen. When the stimulus rate is below the resonance frequency range, the response is weak and shows power at high-frequency harmonics (e.g., the 1 Hz condition in Figure 6B). When the stimulus rate is within the resonance frequency range, a strong response is seen at *f*_0_ and also harmonic frequencies falling into the resonance frequency range (e.g., the 4 and 6 Hz conditions in Figure 6B). Finally, if the stimulus rate is above the resonance frequency range, the steady state response is very weak (e.g., the 12 Hz condition in Figure 6B). Therefore, if strong neural entrainment is seen at *f*_0_ and not at any harmonic frequency, it indicates that the *f*_0_ is within the range of resonance frequency while 2*f*_0_ is outside this range.

What needs additional explanation is why the response is so weak at any frequency when the stimulus rate is low (Figure 6A). When the stimulus rate is below the resonance frequency range, the neural system has no difficulty producing a response for every stimulus event. The reason why the response is weak is two-fold. First, the response only deviates from the baseline in a short period after each stimulus event, making the total power of the response, i.e., power summed over time, very low. Second, the power in the frequency domain is distributed over several harmonic frequencies, making the response at each single frequency even weaker.

In the above discussion, we only consider the neural responses to a sequence of briefly sensory events. Nonetheless, many sensory stimuli, such as speech and music, change continuously. How neural activity follows a continuously changing stimulus is still an unresolved research question (for a review see, Ding and Simon, 2014). One hypothesis is that the response is still triggered by discrete sensory or perceptual events, e.g., acoustic edges or syllable/sentence onsets, in which case the above discussion still holds. The other hypothesis, however, is that the response follows stimulus continuously. Under this hypothesis, for a linear time-invariant system, the response is the continuously changing stimulus feature convolved by the event-related response. If the stimulus feature changes smoothly, e.g., sinusoidally, its power will concentrate at the fundamental frequency. In this case, the response power will also concentrate at the fundamental frequency and any response at harmonic frequencies will reflect nonlinear neural processing.

## Interpreting Low-Frequency Neural Entrainment

When a response shows strong power at *f*_0_, it indicates “baseline” fluctuations within each stimulus period. For example, when *f*_0_ is below 1 Hz and a strong neural response is seen at *f*_0_, it indicates a slow drift in the response “baseline” over the time interval comparable to the duration of a stimulus cycle. If a response is “local”, i.e., lasting for a duration shorter than the stimulus cycle, it can hardly contribute to the neural tracking at the fundamental frequency (Figure 6, 1-Hz condition). For example, the mid-latency auditory evoked response lasts for less than 100 ms. If this response repeats every 1 s, it can hardly contribute to 1-Hz neural entrainment. Very low-frequency (e.g., <1 Hz) neural tracking indicates long lasting and slowly changing responses. The slowness of low-frequency entrainment is in fact its core feature. A key hypothesis about low-frequency neural entrainment is that the neural response does not return to baseline when the next stimulus comes, and this “baseline drift” provides a context for the processing of the next stimulus (Schroeder et al., 2008; Schroeder and Lakatos, 2009).

## Low-Frequency Neural Entrainment During Speech, Music, and Auditory Processing

Low-frequency neural entrainment is often observed during speech and music processing. When listening to discourse-level speech materials, neural entrainment is reliably observed in the delta band (>4 Hz), including the frequency range near or below 1 Hz (Ding and Simon, 2012; Zion Golumbic et al., 2013; Koskinen and Seppä, 2014; Lankinen et al., 2014). It is further demonstrated that neural entrainment in the delta band reflects not only neural encoding of acoustic features but also neural encoding of syntactic structures (Ding et al., 2016). Similar delta-band neural entrainment is observed during music processing. In particular, it has been shown that neural activity can follow the rhythm of musical beat and meter (Nozaradan et al., 2011, 2012; Sturm et al., 2014; Tierney and Kraus, 2014). These results suggest that low-frequency neural entrainment could play a role in parsing the temporal structure of speech and music, forming phrasal-level chunks.

On the other hand, although very low-frequency neural entrainment is reliably observed during speech and music processing, it is not at all a universal phenomenon during the processing of arbitrary auditory stimuli, even ones with a very low-frequency acoustic rhythm (Lakatos et al., 2013; Doelling and Poeppel, 2015). For example, in a study conducted by Lakatos et al. (2013), tone pips are presented every 1.5 s. Only transient auditory evoked responses, i.e., the P1-N1-P2 complex, are seen after each tone pip during passive listening. When the subjects are performing an outlier detection task, however, a slow drift in baseline throughout the 1.5 s stimulus period emerges (in healthy subjects but not in schizophrenia patients). For another example, Doelling and Poeppel (2015) studied the neural responses to music at a very slow tempo, in some cases below 1 Hz. Neural responses of musicians are entrained at the tempo of a given piece while neural responses of non-musicians only appear at harmonics of the tempo rate. Both examples show that very slow neural entrainment (>1 Hz) is not a natural consequences of sensory evoked responses and only emerges as a consequence of task engagement or expertise.

Slow event-related responses such as the N400, P3, and P600 components can indeed contribute to very low frequency neural entrainment near 1 Hz or below. It is usually difficult to dissociate these slow event-related responses from low-frequency neural entrainment simply based on the response spectrum/waveform (O’connell et al., 2012). Nevertheless, the neural entrainment paradigm does not need to isolate the response to a single event and therefore provides a more flexible research paradigm. Furthermore, very low-frequency neural entrainment has been observed in primary sensory areas which are not viewed as the generators of long-latency event-related responses (Lakatos et al., 2008, 2009). To show that entrained activity is distinct from a classic long-latency event-related response, another approach is to show that they have distinct functional properties (Ding et al., 2016).

Therefore, low-frequency neural entrainment at the fundamental frequency of a stimulus rhythm generally implies slow fluctuations in the neural response waveform, which can be viewed as a slow drift in the response “baseline” within each stimulus cycle. Neural activity that oscillates at a rate much faster than the rhythm of the stimulus and transient neural responses that dies off within a small portion of a stimulus period are more strongly reflected by harmonic frequencies in the spectrum. When low-frequency neural entrainment emerges at the fundamental frequency of the stimulus rhythm, it indicates that the influences of previous stimuli do not die out when the next stimulus comes. In other words, previous stimuli set the (neural) context in which a new stimulus will be processed.

In sum, low-frequency neural entrainment implies that either the neural generators have slow intrinsic dynamics or that the neural system continuously tracks certain smoothly changing stimulus properties. It is unlikely that very low-frequency neural entrainment (e.g., <1 Hz) is composed of a series of transient responses evoked by discrete sensory/perceptual events.

## Author Contributions

ND conceived the study. HZ and ND did the simulations. HZ, LM, DP, and ND wrote the article.

## Funding

Work supported by National Natural Science Foundation of China 31500873 (ND), Fundamental Research Funds for the Central Universities (ND), Zhejiang Provincial Natural Science Foundation of China LR16C090002 (ND), and USA National Institutes of Health grant 2R01DC05660 (DP).

## Conflict of Interest Statement

The authors declare that the research was conducted in the absence of any commercial or financial relationships that could be construed as a potential conflict of interest.

## Footnotes

^1^In Figure 5B, we only consider three cycles of the periodic signal, and therefore two zeros are inserted between any two frequencies resolved in Figure 5A (i.e., the frequencies shown by a x in Figure 5A). In general, if the signal contains *N* cycles, *N*−1 points will be inserted between any two frequencies resolved in Figure 5A. If the signal is truly periodic and is infinite in duration, the spectrum is continuous and is zero at any frequency except for the frequencies resolved in Figure 5A.

^2^For linear time-invariant systems, the neural response is the stimulus convolving the event-related response. The Fourier transform of the convolution of two signals is the product of the Fourier transform of each signal. The stimulus considered here is a periodic pulse train. The Fourier transform of a pulse train is also a pulse train. The spectrum of a pulse train is only nonzero at 0, *f*_0_, 2*f*_0_, 3*f*_0_… and take the same nonzero value at 0, *f*_0_, 2*f*_0_, 3*f*_0_… . The Fourier transform of the periodic neural response is the product of the spectrum of the event-related response and the spectral domain pulse train. Therefore, the spectrum of the event-related response is the envelope of the spectrum of the periodic response.
